# Ameliorating Child poverty through Connecting Economic Services with child health Services (ACCESS): study protocol for a randomised controlled trial of the healthier wealthier families model in Sweden

**DOI:** 10.1186/s12889-022-14424-x

**Published:** 2022-11-25

**Authors:** Nina Johansson, Anna Sarkadi, Inna Feldman, Anna M. H. Price, Sharon Goldfeld, Tapio Salonen, Katarina Wijk, David Isaksson, Emir Kolic, Sara Stenquist, Maria Elg, Ewa Lönn, Josefine Wennelin, Linda Lindström, Mirelle Medina, Sofie Åberg, Jessica Viklund, Georgina Warner

**Affiliations:** 1 Child Health and Parenting (CHAP), Department of Public Health and Caring Sciences, Uppsala University, Uppsala, Sweden; 2 Centre for Community Child Health, Murdoch Children’s Research Institute, Royal Children’s Hospital, Melbourne, VIC Australia; 3 Department of Social Work, Malmö University, Malmö, Sweden; 4 Centre for Research and Development, Uppsala University, Gävle, Region Gävleborg Sweden; 5 Faculty of Health and Occupational Studies, Department of Occupational Health Sciences and Psychology, University of Gavle, Gävle, Sweden; 6 Department of Public Health and Caring Sciences, Health Services Research, Uppsala University, Uppsala, Sweden; 7 Konsument Gästrikland Budget and Debt Counselling Service, Gävle, Sweden; 8 Sandviken Municipality, Sandviken, Sweden; 9 Gävle Municipality, Gävle, Sweden; 10 Helsingborg Municipality, Helsingborg, Sweden; 11 Public Contributor, Sandviken, Sweden

**Keywords:** Child poverty, Economic services, Child health services, Healthier wealthier families, Sweden

## Abstract

**Background:**

Sweden is often held up as an example of a country with low child deprivation; yet, rates of relative deprivation are rising. Every municipality in Sweden is required to provide free, timely and accessible budget and debt counselling under the Social Services Act. The services have been encouraged to perform preventative practice with families; however, this has not been realised. The *Healthier Wealthier Families* (HWF) model embeds universal screening for economic hardship into child health services and creates a referral pathway to economic support services. Given the universal child health system in Sweden, which is freely available and has excellent coverage of the child population, implementation of the HWF model has potential to support families to access the freely available municipal budget and debt counselling and ultimately improve rates of child deprivation in Sweden.

**Methods/design:**

We will conduct a two-arm randomised waitlist-control superiority trial to examine the effectiveness and cost-effectiveness of the HWF model in the Sweden. A longitudinal follow-up with the cohort will explore whether any effects are maintained in the longer-term.

**Discussion:**

HWF is a collaborative and sustainable model that could maximise the effectiveness of current services to address child deprivation in Sweden. The study outlined in this protocol is the first effectiveness evaluation of the HWF model in Sweden and is a crucial step before HWF can be recommended for national implementation within the child health services.

**Trial registration:**

Clinicaltrials.gov; NCT05511961. Prospectively registered on 23 August 2022. https://clinicaltrials.gov/ct2/show/NCT05511961

## Strengths and limitations of this study

• This study will assess the effectiveness of the Healthier Wealthier Families (HWF) model on self-reported child deprivation in Sweden by randomised controlled trial
• The study design includes measures on a number of hypothesised mediators, namely self-reported financial knowledge, financial control, readiness for change, parental mental health and financial stigma, which will give insight to the intervention logic model
• Data on income, and sources of income, will allow the amount of financial gain that can be attributed to the HWF model to be described
• Inclusion of personal goal attainment as an outcome will allow participants to express intervention expectations in their own words and report on how far these expectations have been met
• The study design includes a cost-effectives evaluation that will consider group differences in capability-adjusted life-years
• An internal pilot will assess the feasibility of the RCT processes
• A 12-month follow-up will allow for exploration of whether any effects are maintained in the longer-term, albeit without the ability to make group comparisons at that stage

## Background

Sweden is often held up as an example of a country with low child deprivation; yet, rates of relative deprivation are rising. Since 2000, Sweden has witnessed a two-fold increase in the proportion of children living in relative poverty according to the European Union (EU) standard, i.e. living in a household with an income below 60 per cent of the national median [21]. The latest statistics place this figure at 17% [21]. When removing the aspect of relativity and considering families receiving social benefits and/or with a low-income standard, i.e. the household disposable income is lower than considered necessary for living expenses, the most recent figure stands at 9% [19]. Certain families are at greater risk than others. For instance, family constellation affects economic vulnerability, with increased risk of poverty [18] and elevated concerns regarding savings and managing unexpected costs [1] among single-parent households. Parental level of education and immigrant background have also been linked to higher risk of exposure to child poverty in Sweden ([1, 17]).

The impact of relative child poverty has been widely documented, with research reporting negative impacts across various outcomes including mental health problems, obesity, long-standing illness and mortality [10, 22]. The Family Stress Model [4] describes how parental psychological stress can mediate poor child outcomes; economic hardship affects parental mental health, causing parental conflict and/or difficulties with parenting, which in turn negatively affect child outcomes.

Budget and debt counselling, covering aspects such as budgeting, saving and debt management, can offer necessary support to families. Budget and debt counsellors work to improve financial knowledge and enable greater financial control. Not only has the ability to exert control over one’s finances been associated with financial wellbeing, it is actually a stronger predictor than income [23]. In some cases, the ability to meet costs can be improved even in the absence of being able to increase household income, through greater control over the existing income. Every municipality in Sweden is required to provide free, timely and accessible budget and debt counselling under the Social Services Act. The services have been encouraged to perform preventative practice with families; however, this has not been realised. Financial stigma could be playing a role in this. Research shows that Swedish people tend not to share their situation of economic vulnerability with governmental services, even though the services could improve their economic situation [6]. When parents share information about their economic situation, the moral dimension is something that is emphasised. Often, parents want to demonstrate they are financially responsible people, that they prioritise necessary costs and that they put the needs of their children first. Another important aspect to consider is readiness for change. Michie, Atkins, and West [12] describe that in order for behaviour change to take place, one must feel capable, have the opportunity to change, and be motivated to make the change.

**Fig. 1:**
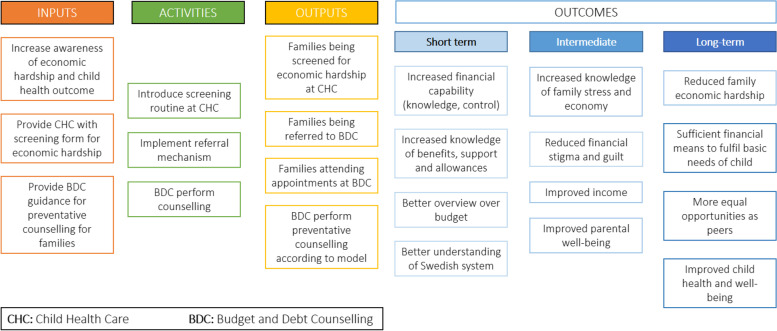
Logic model for Healthier Wealthier Families in Sweden

Systematic integration of financial counselling and income maximisation services into routine health services can provide an opportunity for families to seek support and shift the focus away from the individual, instead emphasising need at the societal level. A recent systematic review identified examples targeting parents of young children across New Zealand, the UK and USA [3]. One such example is the *Healthier Wealthier Children* project, funded by the Scottish Government, which created information and referral pathways between the National Health Service (NHS) early years workforce and financial counselling services [16]. Pre-post evaluation of the model demonstrated monetary gain, as well as improved health, housing and quality of life [16]. The model has been integrated into the Scottish Government policy and adapted to international contexts, including Australia where it has been given the name *Healthier Wealthier Families* (HWF). Whilst the existing evidence is promising, more rigorous evaluation of the model is warranted to evaluate its effectiveness [3]. Given the universal child health system in Sweden, which is freely available and has excellent coverage of the child population [24], implementation of the HWF model has potential to support families to access the freely available municipal budget and debt counselling. This paper sets out the study protocol for a randomised controlled trial (RCT) evaluation of the HWF model in Sweden. The logic model for HWF in Sweden is illustrated in Fig. 1.

### Objectives

The objectives of the trial are:

1. To evaluate whether allocation to municipal budget and debt counselling services via the HWF model and provision of a financial guidance book (intervention arm) has an effect on self-reported child deprivation in comparison to similar families who only receive the financial guidance book (waitlist-control arm).
2. To evaluate whether allocation to the intervention arm has an effect on self-reported financial knowledge, financial control, readiness for change, attainment of personal goals to improve one’s financial situation, parental mental health and financial stigma, which relate to the model theory of change.
3. To describe the amount of financial gain, and sources of income, that can be attributed to the HWF model.
4. To assess whether effects on self-reported child deprivation, financial control, financial knowledge, readiness for change, personal goals, parental mental health and financial stigma are maintained 12 months post-randomisation.
5. To estimate the cost-effectiveness of the HWF model.

It is hypothesised that, when compared with the waitlist-control arm 3 months post-randomisation, families in the intervention arm will report a lower rate of child deprivation, measured as necessary items lacking on a standardised list, due to inability to cover costs. It is further hypothesised that, when compared with the waitlist-control arm, the intervention arm will report greater financial control and knowledge, and lower levels of mental health difficulties and financial stigma.

## Method

### Design

A two-arm randomised waitlist-control superiority trial (1:1 allocation ratio) will be conducted to evaluate the effectiveness of the HWF model in improving the financial situation of families who have self-reported economic difficulties. The intervention arm will be referred to budget and debt counselling immediately after randomisation and the waitlist-control arm 3 months later. Informed by Patient and Public Involvement (PPI), so all participants receive some form of support at randomisation, both arms will be offered a freely available financial guidance book. RCT assessments will take place at two points: pre-intervention (T1) and post-intervention (T2; 3 months after randomisation).

**Fig. 2:**
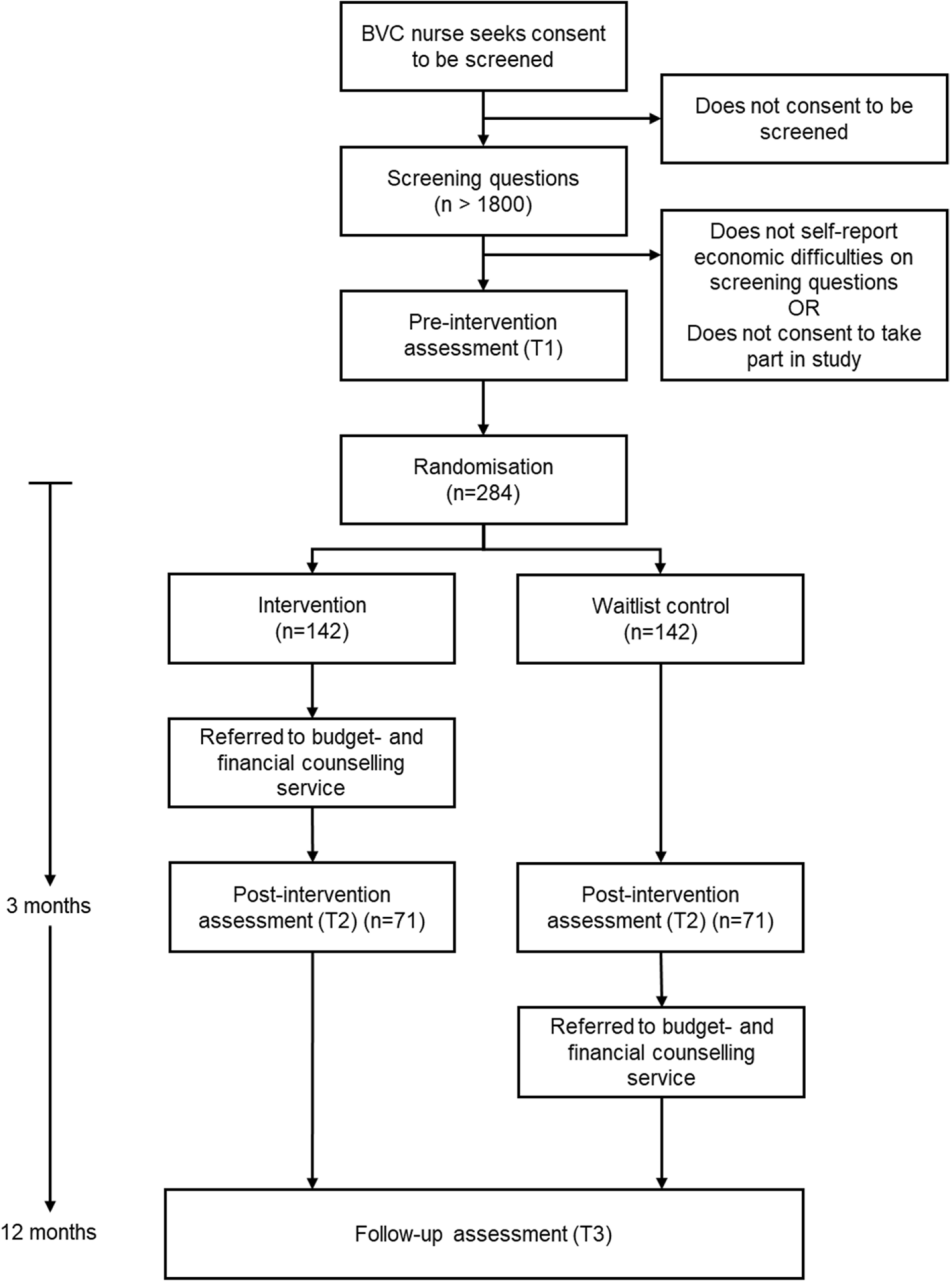
Participant timeline with anticipated numbers

A longitudinal cohort study will be conducted with all participants, from both trial arms (intervention and waitlist-control). Longitudinal assessment will take place 12 months after randomisation (T3), to assess any changes in measures between T2 and T3. See Fig. 2 for the participant timeline.

### Setting

The screening for the RCT will take place at children’s health care centres, known in Swedish as barnavårdscentraler (BVCs). BVCs examine the health of children (0–5 years) to monitor growth and development and provide advice and support to parents, e.g. about the child's development, breastfeeding, food and diseases. Parents are also offered vaccinations for their children at the BVC. Visits take place from just after birth until the child begins school. These services are offered free of charge and achieve excellent reach (99%) [24]. The RCT will focus on BVCs in municipalities experiencing high rates of children living in relative poverty. The 290 municipalities in Sweden have been categorised according to the proportion of children in households with income less than 60 percent of the median [20]. BVCs in the most severe categories (19.7–26.9%; and 27.0–46.0%) will be targeted for participation in the RCT. Concerning the larger municipalities, a more fine-grained categorisation will be conducted using local statistics, to be able to identify the proportion of children in households with income less than 60 percent of the median in residential areas.

### Participants

The target population is families with children in contact with BVCs. To be eligible for the project, the parent or caregiver needs to score on one or more items that indicate economic difficulties and not have been in contact with a financial advisory service in the last month. The screening questions were developed as part of an initial adaptation and pilot study, and include items used by the Swedish Inspection of Finance. The screening includes six items covering financial worry, ability to meet costs, occupation and handling unexpected costs and current use of financial advisory service(s):

1. During the last 12 months, have you been worried that your family will run out of money at the end of the month?
2. During the last 12 months, have you been able to pay current expenses such as rent, bills, insurance?
3. During the last 12 months, have you had the opportunity to buy necessary items such as clothes and food for yourself and your children?
4. Is there at least one person in your household who currently has a paid job?
5. Would you be able to handle an unexpected expense of 20,000 SEK (approx. £1,500) without asking for help or borrowing money?
6. Have you used a financial advisory service within the last month? *(By that we mean if you have had a scheduled meeting with a budget and debt counselor to get help with your economy)*

### Recruitment

During a routine BVC visit, the nurse will ask the parent/caregiver if they consent to answering questions about their economy. If they agree, the child health nurse will systematically work through an electronic screening form. The form has been co-designed with child health nurses, financial counsellors and families. It is hosted on a secure online platform called Research Electronic Data Capture (REDCap), which is specifically geared to support online and offline data capture for research studies and operations. It is fully customizable to meet the needs of this project and allows secure multi-site access with authentication and data logging that meet General Data Protection Regulation (GDPR) regulation requirements. It is a dynamic form that prompts the nurse to show informational material (e.g. a short video about the financial counselling service) at the appropriate moments during the screening discussion. The data are securely transferred to the research group. Basic data are captured for all families screened and personal details shared for those who consent to be contacted about study participation. A member of the research team then contacts the parent/caregiver to complete the informed consent procedure and the study questionnaire, which can be completed via REDCap, on paper, or orally. All parents/caregivers have the right to decline participation in the research study and can still gain access to the municipal budget and debt counselling service.

### Sample size

The primary analysis for the RCT will be a comparison of the unweighted mean number of child material and social deprivation (MSD) items lacked (T2). The target sample size has been calculated based on this primary analysis. The power calculation has been informed by child MSD data for Sweden collected via the European Union Statistics on Income and Living Conditions (EU-SILC) survey, which reports an unweighted mean of 4.5 items lacked among the materially-deprived population. Using a significance level of 0.05 and 80% power, a total of 142 families (71 per group) will be required to detect a 1-item group difference in the unweighted mean number of child MSD items lacked, assuming a standard deviation of 3. As the trial will be conducted in areas with high rates of socioeconomic disadvantage, we estimate that up to 30% of families will experience financial hardship and be eligible for inclusion. The estimated rate of uptake is 50% among eligible families, based on a pilot in the Sandviken municipality, Sweden. Loss to follow-up is projected at 50%. See Fig. 2 for the participant timeline with projected numbers.

### Randomisation

A computer-generated randomisation sequence will be used to assign the participants to the intervention and waitlist-control arms in a 1:1 ratio. Block randomisation will be generated in a computerised randomisation schedule. Separate randomisation lists will be created for each BVC site. Randomisation will take place after pre-intervention data collection. The allocation sequence will be concealed using an online central randomisation service set up and maintained by a professional third party (www.sealedenvelope.com) that will conceal the sequence until group assignment. The randomisation process will require the research team to log into a password-protected website and enter the relevant data of each newly recruited participant in order to receive the allocation. The research team will inform the participant of the randomisation outcome – that they will be referred to the municipal budget and debt counselling straight away or after a period of 3 months.

### Blinding

Randomisation will take place at the research institution, conducted by the research team, directly after T1 data collection. Allocation status will be recorded on the randomisation website, and emailed to the research team member conducting the randomisation (NJ) and the trial manager (GW). Participants will not be blinded to group allocation, and data collection will not be blinded. Given that neither participants nor budget and debt counsellors are blinded, there is no requirement for an unblinding procedure. Questionnaire data spreadsheets will use participant identity numbers; however, group status will be apparent due to the inclusion of counselling session attendance data for the intervention group.

### Intervention arm

*All* participants will be given a copy of *‘Your child, your money’*, a freely available financial guidance book for new parents at the time of screening. The book has been developed by Finansinspektionen, Sweden's financial supervisory authority, and covers topics such as planning finances, consumer rights, private insurance, family law, parental leave, pensions, saving, and budgeting. (see Table 1 for an overview of content). Participants randomised to the intervention arm will be immediately referred to the local budget and debt counselling service. After receiving the participants’ details, first contact from a budget and debt counsellor will take place within a few weeks. Budget and debt counselling will involve at least one meeting with a counsellor. Examples of assistance the counsellors can offer include: suggestions on ways to improve a participant’s financial situation; checking eligibility and helping to apply for social welfare support; helping to organise finances and develop a budget; and assistance with debt management, threatening letters or harassment by debt collectors, or imminent house eviction. The municipal budget and debt counselling services involved in the trial will receive guidance on how to work preventatively with families, which prompts discussion on needs, personal goals, financial behaviour and income maximisation. Participants may end counselling at any time, and there will be no restrictions on access to other support during the study period.

**Table 1:** Overview of’Your child, your money’ content

Pregnant	Parent	Family
- Plan your economy - Plan for new costs - Your rights as a consumer - Private insurance - Family legislation	- Parental leave - Our pension system - Parental leave and studies - Child insurance - To save for children	- Child sickness - Links - My monthly budget

### Control arm

As described above, all participants receive a copy of the book. Those randomised to the waitlist control arm will be referred to the local financial counselling service after a period of 3 months.

### Participant timeline

Figure 2 provides an overview of the participant timeline. Parents/caregivers are screened for eligibility at a routine BVC appointment. Screening data will be automatically transferred to the research team via a secure online platform. When a parent screens positive and indicates they are interested in participating in the project, a member of the research team will contact them to complete the informed consent procedure and the study questionnaire. A case will be randomised once all pre-intervention data has been collected. Follow-up data will be collected from all participants at scheduled group meetings at two points: 3 months after randomisation (T2) and 12 months after randomisation (T3).

### Measures

**Fig. 3:**
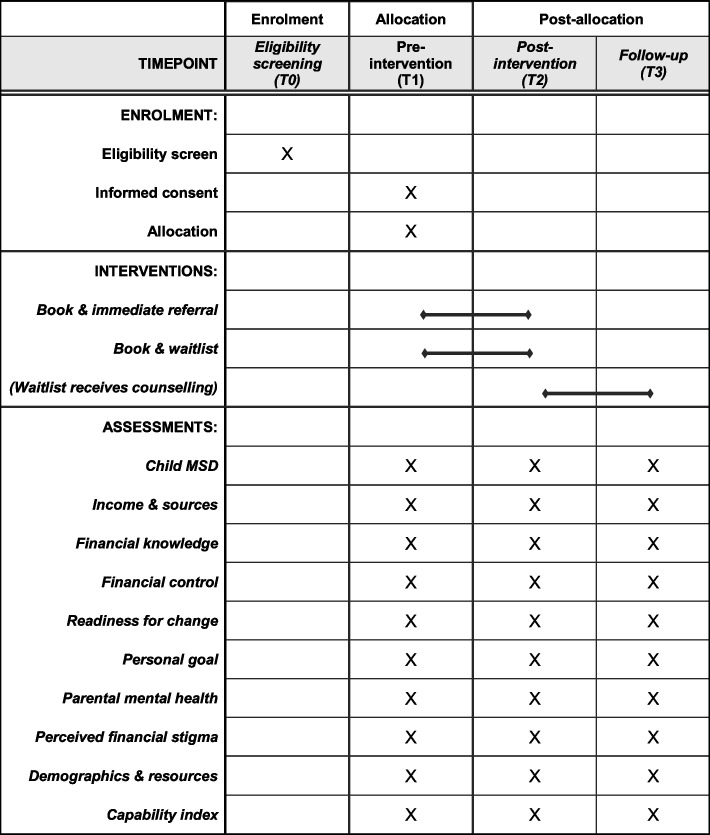
Schedule of enrolment, interventions and assessments

The primary outcome measure for the RCT is child material and social deprivation (MSD). Secondary measures include household income and sources of income, financial knowledge, financial control, readiness to change, personal goal attainment, parental mental health, and financial stigma. These measures will be administered at all time points and assessed at T2 (for the RCT) and T3 (for the longitudinal cohort study). See Fig. 3 for an overview of the enrolment, intervention and assessment schedule.

Due to the target population being both Swedish speaking and non-Swedish speaking, there will be translated measures available in the data collection process. Translated measures will be provided for the larger language groups at each project site. For some measures, there are translated versions readily available. For the other measures, we will use a certified translation service. For validation, back-translations will be conducted.

#### Child MSD

The European Union (EU) measure of child MSD [8] contains 17 items; 13 child-specific items (e.g. *Two pairs of properly fitting shoes; Celebrations on special occasions*) and 4 household items (e.g. *Home adequately warm; Access to the Internet*). Parents are asked to indicate whether or not they have the item. If not, they are asked if it is because they cannot afford it (enforced lack) or for another reason (simple lack). Data are collected at the household level; if one child does not have an item it is assumed that all the children in the household lack that item. Cronbach’s alpha indicates adequate reliability of the ‘enforced lack’ concept in Sweden (α = 0.76) and across EU countries (α = 0.76–0.94) [8]. The items have been shown to be additive, i.e. a household with a score of “2” is in reality suffering from more severe MSD than a household with a score of “1” or a score of “0” [8]. As recommended by Gui et al. (2018), an unweighted sum of the 17 MSD items will be calculated for each household in the trial. The primary analysis for the RCT will be a comparison of the unweighted mean number of child MSD items lacked at T2. A secondary outcome of the trial will be a comparison of the proportion of children classified as experiencing deprivation at the 3-month follow-up. As reported by Gui et al. [8], a threshold of 3 items will be applied to the data. In other words, a household lacking two or less items will be classified as ‘*not* experiencing deprivation’ and a household lacking three or more items will be classified as ‘experiencing deprivation’. Both the unweighted mean number of child MSD items lacked and the proportion of children classified as experiencing deprivation will also be assessed at T3 as part of the longitudinal follow-up.

#### Household income and sources of income

Participants will be asked to report their overall household income on an incremental scale up to the median salary for Sweden, as well as their fixed outgoings (e.g. rent and bills) to enable calculation of the net income for the household. They will also be asked to select all applicable sources of income from a list, which includes all available benefits in Sweden.

#### Financial knowledge

A selection of questions covering financial knowledge (e.g. invoice payment periods, mortgages, debt collection) and where to turn for help for better control over finances and debt management will be taken from a survey conducted by the Swedish Consumer Agency survey [9].

#### Financial control

Respondents will be asked to indicate the extent to which they agree or disagree with a series of nine statements (e.g. *My financial situation is largely outside of my control*) using a 5-point scale, ranging from 1 (strongly disagree) to 5 (strongly agree).

#### Readiness for change

To assess readiness for change, participants will be asked to complete a Readiness Ruler [14] regarding changing their financial situation. The ruler will employ a 0–10 visual analogue scale. Scores of 1–3 represent non-readiness to change, scores of 4–6 uncertainty, scores of 7–8 represent readiness, and 9–10 represent ongoing attempts at changing.

#### Personal goal

Participants will be asked to write a personal goal for improving their financial situation (*Over the next 3 months, what is your personal goal for improving your financial situation?*) at T1. Their own words will be presented back to them at T2 and T3, and they will be asked to rate how far they have achieved their goal (*On a scale of 0–10, how far have you achieved your goal: [parent’s own words inserted here]*?) A visual analogue scale ranging from anchors “not at all” for the value 0 to “extremely” for the value 10 will be used.

#### Parental mental health

The 12-Item General Health Questionnaire (GHQ-12) [7] consists of 12 items (e.g. *Able to concentrate, Loss of sleep over worry, Capable of making decisions*). Respondents are asked to rate the degree to which they have experienced a symptom during the last week with four response categories (e.g. *Less than usual, No more than usual, Rather more than usual, or Much more than usual*. The questionnaire primarily includes depression symptoms, but also some anxiety symptoms. The minimum score is 0, and the maximum is 36. A higher score indicates a higher level of distress. The Swedish version of the GHQ-12 performed excellently in a case–control study assessing discriminant validity (sensitivity = 85.5; specificity = 83.2 [11].

#### Perceived financial stigma

An 8-item measure of perceived financial stigma will be used [13]. Respondents are asked to indicate the extent to which they agree or disagree with a series of eight statements using a 5-point scale, ranging from 1 (*Definitely disagree*) to 5 (*Definitely agree*). The items cover two dimensions of perceived stigma (each consisting of four items): internalized stigma (e.g., *I feel that I am odd or abnormal because of my financial situation*) and experienced stigma (e.g., *I feel that others look down on me because of my financial situation*).

#### Demographic information and consumption of societal resources

The study will gather demographic information about the parent and their child(ren), including variables such as employment status, child age, gender. Based on previous research [1, 17], we anticipate an over-representation of participants with cognitive difficulties, non-Swedish speaking participants and single caregiver households. Due to this, demographic questions on disabilities in the household, native language and living arrangements will be included. The demographic data will be used to describe the trial sample, examine the extent to which demographic characteristics are balanced between trial arms, carry out attrition analyses, and inform the cost-effectiveness evaluation. The demographics questions will be administered at T1. Items for which the response may change (e.g. employment status, contact with health care professionals) will be administered at T2 and T3.

#### Capability index

A 6-item measure of capability [15] will be used to estimate capability adjusted life years (CALY) for the cost-effectiveness evaluation. Respondents are asked to indicate whether they totally agree, partly agree, or do not agree with a series of six statements. The items cover health, occupation, social relations, security, and political and civil rights, and financial situation (e.g. *I have an economy (salary, other income or savings) that allows me to always have a permanent home and for the most part (at least 8 times out of 10) allows me to buy what I think I need*). These questions will be administered at all time points and assessed at T2 and T3.

#### Intervention fidelity

Session attendance and the topics covered during sessions will be recorded by the budget and debt counsellors on a brief fidelity form, which will be shared with the research team to inform individual participant dose and adherence to the preventative family counselling guidance. Further to this, participant identification numbers will be checked against budget and debt counselling records. This process will identify any group contamination within the trial i.e. participants allocated to the intervention arm that do not receive counselling, or participants allocated to the waitlist control that do receive counselling.

### Data collection

T1 data collection and randomisation is planned to take place between September 2022 and June 2024. T2 data collection occurs around 3 months after T1 data collection, and is therefore due to take place between December 2022 and September 2024. T3 data collection occurs 12 months after T1 data collection and is due to take place between September 2023 and June 2025.

Data will primarily be collected using REDCap. The study questionnaire can be completed via an electronic link. Alternatively, it can be completed over the phone with a member of the research team, or on paper via post. This inclusive data collection practice allows for participant preferences to be taken into account and overcomes potential literacy difficulties that could be present in the target population. The screening form includes a question on need for interpretation, which will be consulted prior to the research team contacting the parent/caregiver to allow for necessary interpreter arrangements to be made. Participants will be offered shopping vouchers at T2 and T3 to compensate for their time completing the study questionnaire. Data will be exported into the Statistical Package for the Social Sciences (SPSS) for analysis. Participant identity numbers will be used. The file will be saved on the university server, which is automatically backed up. All data management procedures comply with current regulations on personal data management.

### Statistical methods

Baseline and demographic characteristics will be summarised using means and standard deviations (or medians and interquartile ranges) for continuous variables and percentages for categorical variables. For items that align with national survey questions, population-level comparisons will be made. Descriptive data will also be reported on process outcomes, e.g. number of families screened, proportion eligible for inclusion, and proportion consenting to participate.

The primary comparison of the trial arms will use an intention-to-treat framework. A regression analysis will be undertaken to quantify the extent to which a potential intervention effect on the primary outcome is determined by allocation to the intervention. Per-protocol comparison will be performed as a secondary analysis. Further moderation analyses will examine the associations between outcomes and participants’ characteristics. Differences in the means and proportions on measures between T2 and T3 will be assessed using parametric and non-parametric tests.

The cost-effectiveness analysis will compare the two arms of the trial at T2 follow-up using capability-adjusted life-years (CALY) gained as the outcome of interest. To analyse the cost differences between the two arms, we will collect information about intervention costs (i.e. personnel time, material, etc.) and societal resource use. We will express the final output as a cost/ CALY. Results will be presented as incremental cost-effectiveness ratios (ICER, expressing the cost per additional CALY gained). Uncertainty will be demonstrated using a cost-effectiveness acceptability curve, using bootstrapped regression estimates [5].

### Internal pilot

An internal pilot RCT will be conducted. The target N is 20 eligible families (10 per arm). The primary objective of the pilot study is to assess the feasibility of the RCT processes as a qualitative pilot evaluation has already taken place. Effectiveness will not be evaluated at this stage, but descriptive statistics will be reported for the trial measures, as well as adherence rates and reasons for non-adherence. A process for Decision-making after Pilot and feasibility Trials (ADePT) [2] will be used to support systematic decision-making in moving forward with the trial.

## Discussion

HWF is a collaborative and sustainable model that could maximise the effectiveness of current services to address child deprivation in Sweden. The study outlined in this protocol is the first effectiveness evaluation of the HWF model in Sweden and is a crucial step before HWF can be recommended for national implementation within the child health services. However, several operational challenges are anticipated. Given that parental level of education and immigrant background have been linked to higher risk of exposure to child poverty in Sweden, the accessibility of the study questionnaire is a potential issue. The trial will operate across multiple sites and there is likely to be variation in the municipal budget and debt counselling services. As the services are freely available and open for self-referral there is also relatively high risk for trial arm contamination. However, we have put actions in place to have oversight of and try to mitigate against these risks. The study information materials and questionnaire will be made available in the languages commonly spoken in the targeted areas, and data collection can take place online, by post, or via phone. Brief counselling guidance has been developed specifically for the HWF project and will be provided to all trial sites in an effort to standardise the intervention, without compromising the personalised nature of budget and debt counselling. A fidelity checklist will be administered to record adherence to the guidance topics. Further to this, participant identification numbers will be checked against budget and debt counselling records to identify trial arm contamination.

## Data Availability

The results from the ACCESS internal pilot are due to be submitted for publication in 2023, the RCT results in 2025 and the longitudinal follow-up in 2026. Authorship will be granted for substantive contributions to the design, conduct, interpretation and reporting of the ACCESS trial; the ultimate decision on authorship will be made by the Principal Investigator (AS). Publications will be open access. The datasets generated during the current study will be available from the Principal Investigator (AS) on reasonable request.

## Abbreviations

ACCESS: Ameliorating Child poverty through Connecting Economic Services with child health Services
ADePT: A process for Decision-making after Pilot and feasibility Trials
BVC: Barnvårdscentral / child health care centre
CALY: Capability-adjusted life-years
EU: European Union
GDPR: General Data Protection Regulation
HWF: Healthier Wealthier Families
ICER: Incremental cost-effectiveness ratios
MSD: Material and social deprivation
NHS: National Health Service
RCT: Randomised controlled trial
REDCap: Research Electronic Data Capture
